# Hemolytic Anemia From Native Aortic Valve Infective Endocarditis Due to Streptococcus gordonii in a Patient With End-Stage Renal Disease on Hemodialysis: A Case Report

**DOI:** 10.7759/cureus.108273

**Published:** 2026-05-04

**Authors:** Dung Cindy H Nguyen, Wissam A Saliba

**Affiliations:** 1 Department of Internal Medicine, University of Kansas School of Medicine Wichita, Wichita, USA; 2 Nephrology, Ascension Via Christi St. Francis Hospital, Wichita, USA

**Keywords:** end-stage renal disease, hemodialysis, hemolytic anemia, infective endocarditis, mechanical hemolysis, native aortic valve endocarditis, native valve hemolytic anemia, severe aortic regurgitation, streptococcus gordonii, viridans group streptococci

## Abstract

Infective endocarditis (IE) complicated by non-immune hemolytic anemia is a rare but clinically significant presentation, particularly in native valve disease, and often reflects severe valvular destruction leading to mechanical red blood cell fragmentation. Patients with end-stage renal disease (ESRD) on hemodialysis are at increased risk of bloodstream infections due to chronic vascular access, making early recognition essential.

We report the case of a 36-year-old man with ESRD on hemodialysis via an arteriovenous (AV) graft who presented with acute dyspnea, hypoxia, and severe transfusion-refractory anemia. Laboratory evaluation revealed non-immune hemolytic anemia consistent with mechanical destruction. Blood cultures grew Streptococcus gordonii. Transesophageal echocardiography confirmed IE, demonstrating aortic valve vegetations with severe aortic regurgitation. His hospital course was further complicated by community-acquired pneumonia and acute hypoxic respiratory failure. The hemolytic process resolved after management with targeted intravenous antibiotics and urgent bioprosthetic aortic valve replacement.

This case highlights the importance of considering subacute IE in patients with ESRD who present with persistent, unexplained hemolytic anemia, particularly in the setting of chronic vascular access. Early diagnosis and prompt intervention are critical to reducing morbidity and mortality associated with this uncommon but life-threatening complication.

## Introduction

IE constitutes a severe and often fatal complication among patients with end-stage renal disease (ESRD) receiving maintenance hemodialysis. The heightened vulnerability in this population arises from repeated vascular access and uremia-induced immune dysfunction, both of which predispose to bacteremia and subsequent endocardial seeding [1]. Consequently, individuals undergoing long-term hemodialysis have a higher incidence of IE, increased in-hospital mortality, and lower rates of surgical valve intervention compared with the general population [2,3].

Anemia is a common comorbidity in ESRD and is also frequently observed in IE. While hemolytic anemia is well described in prosthetic valve dysfunction, it is exceedingly uncommon in native valve endocarditis, with only a limited number of reported cases [4,5].

In patients with ESRD on hemodialysis, Staphylococcus aureus is the leading cause of IE, reflecting the high prevalence of catheter- and vascular access-related bacteremia. Viridans group streptococci account for a smaller proportion of cases in this population. Among them, Streptococcus gordonii, a typically benign, alpha-hemolytic commensal of the oral cavity, is a less prevalent but notable cause of IE [6,7]. It can become pathogenic when mucosal barriers are disrupted, particularly after dental procedures. S. gordonii endocarditis has been associated with embolic complications and severe valvular damage requiring surgical intervention, likely related to its capacity for biofilm formation and the expression of distinct virulence factors [8].

We present an atypical case of native aortic valve infective endocarditis (IE) caused by S. gordonii in a patient with ESRD on hemodialysis via an arteriovenous (AV) graft, complicated by severe nonimmune hemolytic anemia.

This case report was previously presented in poster form at the Kansas American College of Physicians Poster Competition on October 16, 2025.

## Case presentation

A 36-year-old man with ESRD on maintenance hemodialysis via a left thigh AV graft, chronic anemia, prior renal transplant rejection, and poorly controlled hypertension presented with worsening dyspnea and hypoxia. In the preceding weeks, he experienced persistent symptomatic anemia, with hemoglobin levels of 6-7 g/dL despite multiple interventions, including three packed red blood cell (PRBC) transfusions, intravenous iron, and high-dose erythropoietin therapy, suggesting anemia beyond his ESRD baseline. On the day of admission, his pre-dialysis hemoglobin declined to 5.4 g/dL, prompting transfusion of one unit of PRBCs in the emergency department, after which he was discharged.

**Figure 1:**
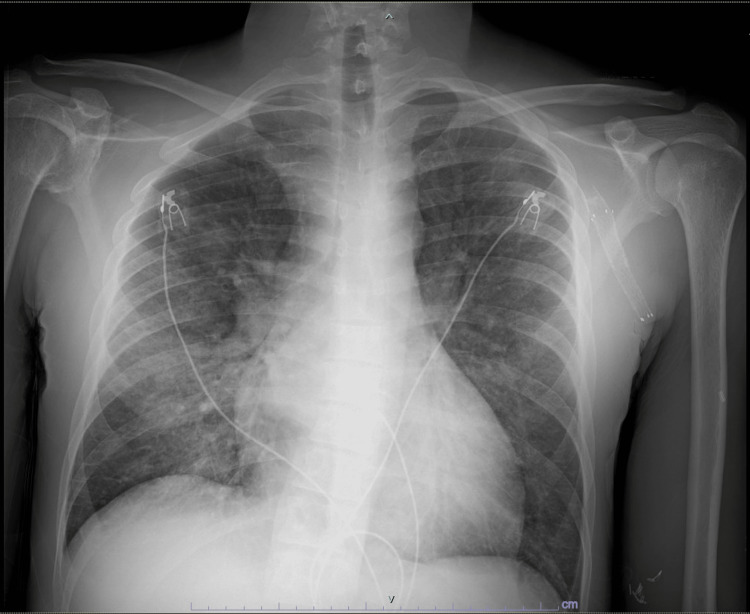
Chest radiograph (frontal view) demonstrating a right perihilar airspace opacity concerning for infection.

He returned shortly thereafter with worsening dyspnea, tachypnea, and tachycardia, necessitating hospital admission. At presentation, he was febrile to 38.1 °C and hypertensive (185/100 mmHg). Relevant laboratory findings are shown in Table 1. Chest radiography demonstrated a right perihilar opacity concerning for pneumonia (Figure 1). Physical examination revealed diffuse crackles and a new grade II/VI systolic murmur, without peripheral stigmata of IE. There was no scleral icterus or jaundice. Transfusion-related acute lung injury was considered but ultimately excluded.

**Table 1:** Relevant lab findings on admission. WBC, white blood cell count; LDH, lactate dehydrogenase; TIBC, total iron-binding capacity

Test	Result	Reference range
WBC (×10³/µL)	20.1	4.8-10.8
Neutrophils (%)	81	51-75
Hemoglobin (g/dL)	6.4	14-18
Hematocrit (%)	22	42.0-52.0
Platelets (×10³/µL)	127	150-400
Reticulocyte (%)	4.7	0.6-2.5
Lactic Acid (mmol/L)	3.5	0.5-2.2
LDH (U/L)	418	125-220
Haptoglobin (mg/dL)	<8	14-258
Total Bilirubin (mg/dL)	1.5	0.1-1.2
Iron (µg/dL)	71	65-175
Ferritin (ng/mL)	1,842	22-275
Transferrin (mg/dL)	134	174-364
Iron Saturation (%)	36	11-46
TIBC (µg/dL)	200	268-490
Vitamin B12 (pg/mL)	1,367	213-816
Folate (ng/mL)	14.2	7.0-31.4

**Figure 2:**
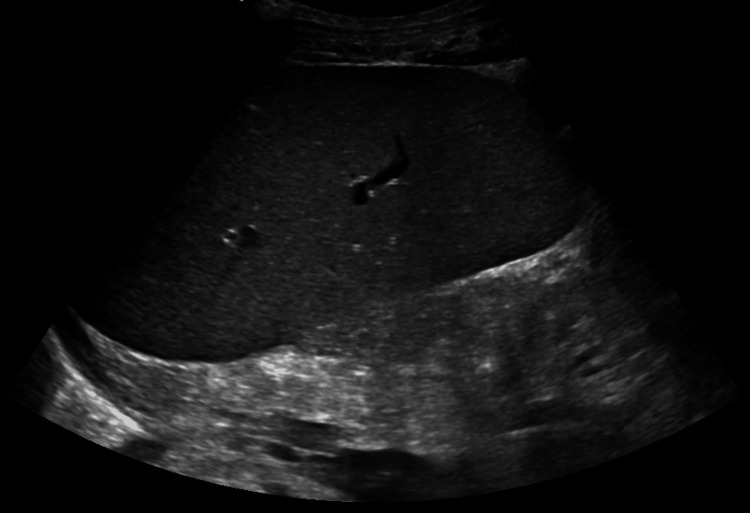
Abdominal ultrasound demonstrating splenomegaly (15.2 x 7.2 x 7.5 cm).

The patient was diagnosed with community-acquired pneumonia and sepsis and empirically treated with ceftriaxone, azithromycin, and vancomycin. Blood cultures subsequently grew S. gordonii in both sets. Based on susceptibilities, therapy was de-escalated to ceftriaxone 2 g daily. Peripheral smear revealed normocytic, non-immune hemolytic anemia with polychromasia, anisocytosis, and schistocytes, along with leukocytosis and thrombocytopenia. Given the constellation of low haptoglobin, schistocytes, elevated LDH, and severe anemia, a workup for hemolysis was pursued. The indirect Coombs test was negative. Abdominal ultrasound demonstrated splenomegaly (Figure 2), likely due to reactive enlargement from increased splenic clearance of fragmented erythrocytes. There was no personal or family history of hereditary hemolytic disorders.

**Figure 3:**
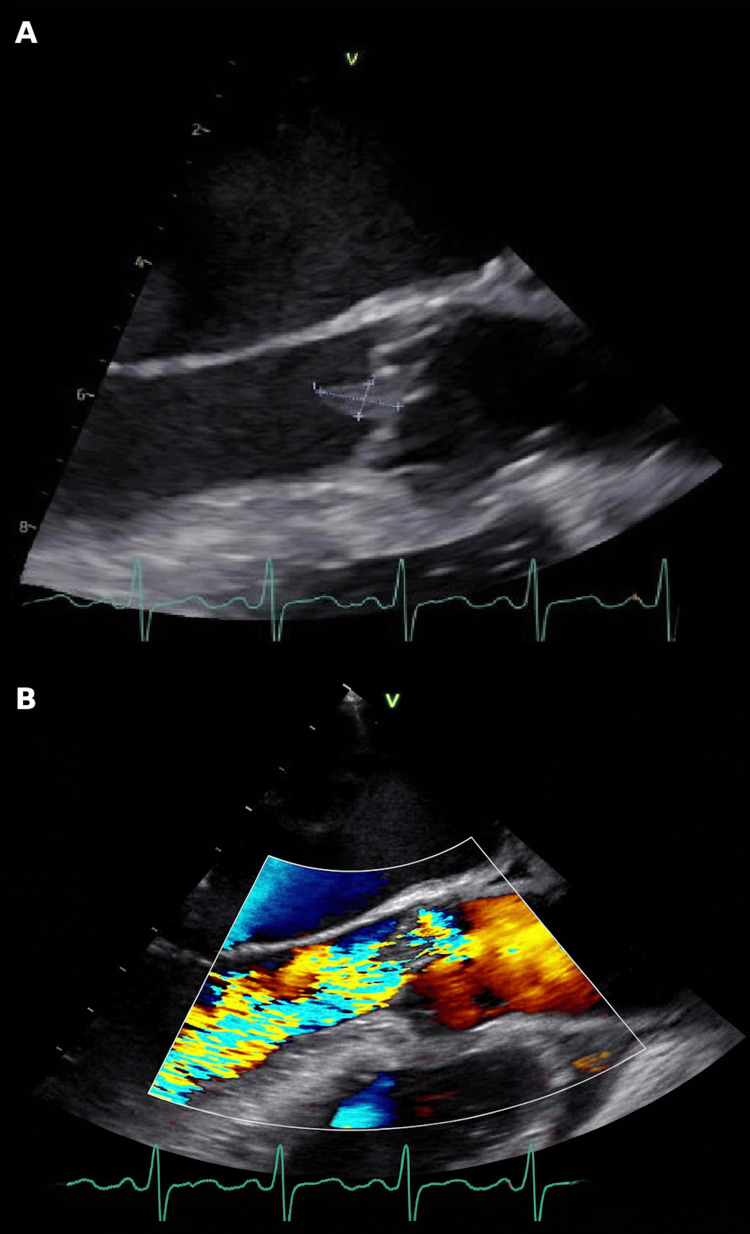
Transesophageal echocardiogram. (A) Mid-esophageal long-axis view demonstrating a 1.0 × 0.5 cm mobile vegetation on the ventricular surface of the left coronary cusp of the aortic valve. (B) Color Doppler imaging demonstrating severe aortic regurgitation.

**Figure 4:**
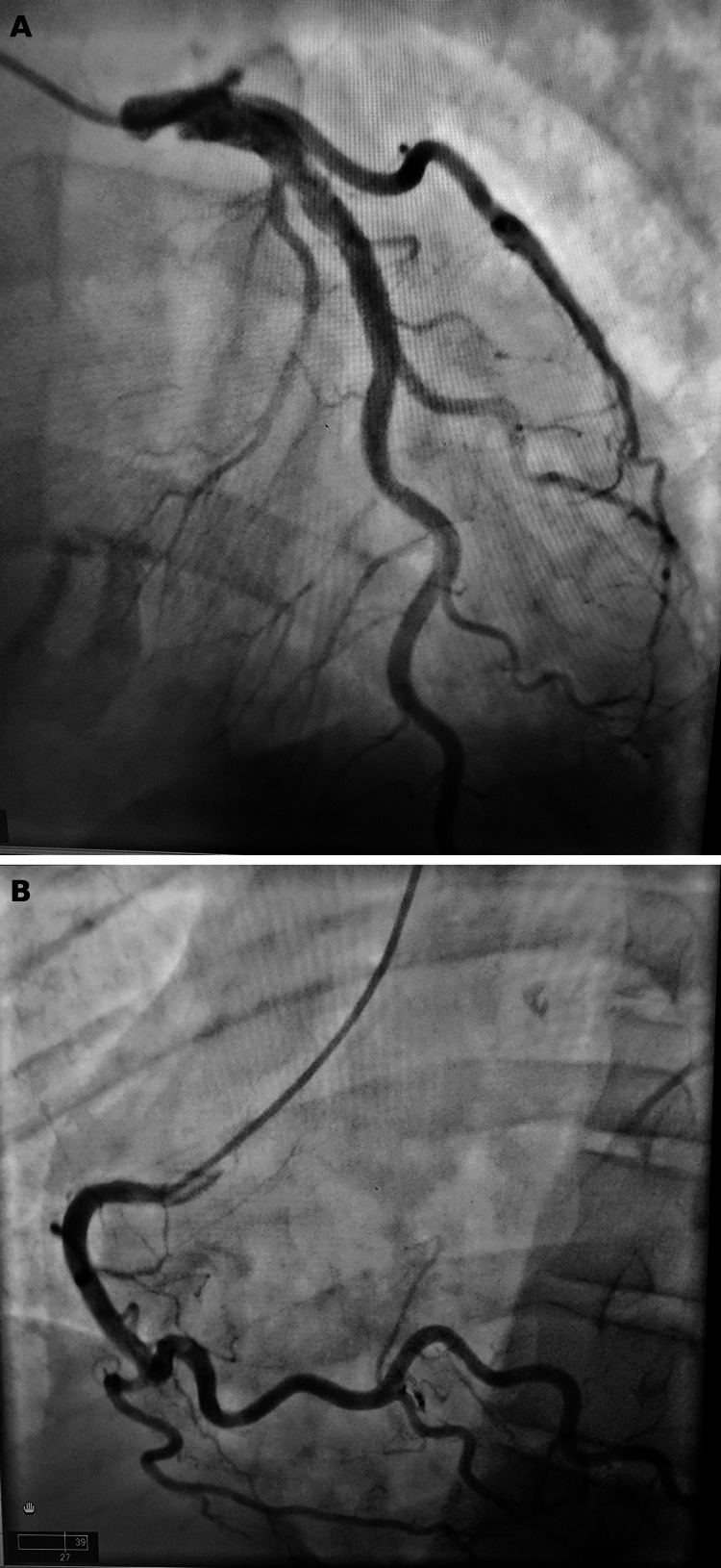
Coronary angiography. (A) Left main coronary artery with no significant obstructive coronary artery disease. (B) Right coronary artery with no significant obstructive coronary artery disease.

Transesophageal echocardiography (TEE) revealed a 1.0 × 0.5 cm mobile vegetation on the ventricular surface of the left coronary cusp of the aortic valve, with severe aortic regurgitation (Figures 3A-3B). Coronary angiography demonstrated no obstructive coronary artery disease (Figures 4A-4B). These findings supported nonimmune mechanical hemolysis in the setting of IE. 

The infectious disease and cardiothoracic surgery teams concluded that the valvular infection was most likely causing mechanical hemolysis due to red blood cell fragmentation across the infected, regurgitant aortic valve. Surgical intervention was advised, with mechanical aortic valve replacement (AVR) favored given the patient’s age (<50 years) and ESRD. However, the patient declined a mechanical prosthesis to avoid lifelong anticoagulation with warfarin. After shared decision-making, he underwent bioprosthetic AVR with implantation of a 23 mm Edwards RESILIA tissue valve (Edwards Lifesciences, Irvine, CA, USA) and aortic root enlargement via the Bo Yang Y-incision annuloplasty technique using a PhotoFix bovine pericardial patch (CryoLife, Kennesaw, GA) [9]. 

Follow-up blood cultures at 48 hours were negative, confirming microbiologic clearance. The patient required a total of two units of PRBCs during hospitalization. Hemodialysis was maintained throughout his inpatient course. Although ceftriaxone is a preferred agent for viridans streptococcal endocarditis, the patient was transitioned to vancomycin to facilitate outpatient management, as vancomycin can be administered after hemodialysis sessions (3x weekly), whereas ceftriaxone typically requires daily dosing. 

Review of his medical and dental history revealed no recent dental procedures or identifiable oral source. The left thigh AV graft, placed two years prior after multiple failed upper-extremity accesses, was considered a possible nidus for bacteremia. However, there was no clinical or imaging evidence of access-site infection, and hemolysis resolved after valve replacement, supporting the valve as the primary source. Because removal of a long-standing, functional AV graft carries significant morbidity and would limit future dialysis access options, the multidisciplinary team favored graft preservation with close surveillance. 

Postoperatively, his hemoglobin stabilized without further transfusion requirements, and his erythropoietin dose was reduced to the minimal effective level, consistent with resolution of hemolysis. 

## Discussion

This case highlights an unusual but clinically important cause of profound anemia in the ESRD population: nonimmune mechanical hemolysis secondary to native-valve IE. While mechanical hemolysis is well recognized in prosthetic valve dysfunction, it is infrequently reported in native-valve disease. In this patient, the mechanism was most consistent with increased shear stress from aortic valve destruction and severe regurgitation [5].

S. gordonii, an oral commensal, can cause IE through platelet-binding adhesins and biofilm formation [6-8]. Although no oral source was identified, the patient’s long-standing AV graft represents a potential but unproven nidus for bacteremia. 

Patients with ESRD experience worse outcomes than the general population, with higher rates of in-hospital and long-term mortality, greater complication burdens, and a higher likelihood of recurrent IE [10]. Hemodialysis access infections remain an important source of morbidity in ESRD and substantially increase the risk of bacteremia and subsequent endocardial involvement. AV grafts that have been in place for many years, are calcified, or are located in the lower limbs present a higher risk due to repeated use and their synthetic surfaces [11].

Anemia in ESRD is typically multifactorial, often attributed to erythropoietin deficiency, iron dysregulation, or uremic toxins. However, this patient’s presentation, marked by schistocytosis, elevated lactate dehydrogenase (LDH), low haptoglobin, and severe anemia, supported a hemolytic process. The absence of a positive Coombs test and lack of clinical evidence for hemoglobinopathy or nutritional deficiency made immune-mediated and hereditary causes unlikely. The history of transfusion-refractory anemia in the weeks preceding diagnosis suggests that hemolysis may have been an early manifestation of evolving valvular pathology.

Early echocardiographic assessment is crucial for dialysis patients presenting with sepsis, unexplained anemia, or laboratory evidence of hemolysis. Viridans group streptococci, such as S. gordonii, can produce rapidly progressive and destructive valvular infections if not promptly identified [7, 12].

Patients on chronic hemodialysis who develop IE have significantly higher one-year and five-year mortality compared with non-dialysis patients [13]. Therefore, collaboration among nephrology, infectious diseases, cardiology, and cardiothoracic surgery is critical for timely diagnosis and coordinated management. Despite higher baseline operative risks, multiple studies suggest that surgical intervention can offer a meaningful survival benefit in ESRD patients when used for appropriate indications, such as valvular destruction or hemodynamic compromise [10,14,15]. A recent systematic review and meta-analysis demonstrated lower 30-day mortality with surgical management, with in-hospital benefits reaching statistical significance after excluding small-sample studies [15]. In this case, resolution of hemolysis following aortic valve replacement further supports a mechanical etiology.

## Conclusions

Hemolytic anemia associated with native-valve IE is exceedingly rare, and S. gordonii represents an uncommon yet potentially aggressive etiologic agent. For patients with ESRD on hemodialysis, this case emphasizes the importance of broadening the differential diagnosis for persistent, transfusion-refractory anemia to include IE when anemia remains unexplained after more common causes have been excluded. Improving survival in this high-risk population requires early echocardiographic assessment and a coordinated multidisciplinary approach, integrating targeted antimicrobial therapy with timely surgical intervention to reverse the hemolytic process and restore hemodynamic stability. In dialysis patients, transfusion-refractory anemia with evidence of hemolysis should prompt immediate evaluation for infective endocarditis.
